# Facile Synthesis, Characterization, and Cytotoxic Activity of Europium-Doped Nanohydroxyapatite

**DOI:** 10.1155/2016/1057260

**Published:** 2016-11-14

**Authors:** Paulina-Guadalupe Miranda-Meléndez, Gabriel-Alejandro Martínez-Castañón, Nereyda Niño-Martínez, Nuria Patiño-Marín, Miguel-Ángel Casillas-Santana, Brenda-Erendida Castillo-Silva, Facundo Ruiz

**Affiliations:** ^1^Doctorado Institucional en Ingeniería y Ciencia de los Materiales, Universidad Autónoma de San Luis Potosí, Av. Salvador Nava s/n, Zona Universitaria, 78290 San Luis Potosí, SLP, Mexico; ^2^Doctorado en Ciencias Odontológicas, Facultad de Estomatología, Universidad Autónoma de San Luis Potosí, Av. Dr. Manuel Nava 2, Zona Universitaria, 78290 San Luis Potosí, SLP, Mexico; ^3^Facultad de Ciencias, Universidad Autónoma de San Luis Potosí, Lateral Av. Salvador Nava s/n, Zona Universitaria, 78290 San Luis Potosí, SLP, Mexico

## Abstract

The objective of this study was to synthetize europium-doped nanohydroxyapatite using a simple aqueous precipitation method and, thereafter, characterize and impregnate selected samples with 5-fluorouracil in order to explore the properties and the releasing capacity of this material. The nanohydroxyapatite was doped with 3, 5, 10, and 20 wt% of europium. The obtained samples were characterized after they were dried at 80°C and hydrothermal treated at 120°C by 2 hours. The samples were analyzed by transmission electron microscopy, X-ray diffraction analysis, Fourier transform infrared spectroscopy, and photoluminescence. Also, impregnation and release of 5-fluorouracil were assessed in PBS. The toxicity effects of all samples were studied using viability assays on human fibroblasts cells (HGF-1)* in vitro*. The sizes of the crystallites were about 10–70 nm with irregular morphology and present the phase corresponding to the JCPDS card 9–0432 for hydroxyapatite. The results of the toxicity experiments indicated that doped and undoped powders are biocompatible with fibroblasts cells. Hydroxyapatite samples doped with 5% of europium and loaded with 5-fluorouracil release almost 7 mg/L of the drug after 60 minutes in PBS and decrease the viability of HeLa cells after 24 hours.

## 1. Introduction

The growing interest in nanostructured materials over the last years is a result of their irreplaceable role in biomedical applications [1]. Hydroxyapatite (HAP), with a composition of stoichiometric Ca_10_(PO_4_)_6_(OH)_2_ and a ratio of Ca/P = 1.67, is the principal inorganic constituent of teeth [2, 3] and is chemically and structurally similar to the mineral portion of bones [4]. HAP has been investigated as a drug delivery system for a variety of pharmaceutical molecules because of their biocompatible, osteoconductive, nontoxic, and noninflammatory properties. It has been shown that hydroxyapatite nanoparticles (nHAP) by themselves have an inhibitory action against different kinds of neoplasia [5, 6].

Nowadays, the synthesis and discovery of multifunctional nanostructured systems hold a promise for the future of medical treatments to improve therapeutic efficiency. It is highly desirable to develop new multifunctional nanostructured systems that could achieve simultaneous* in vivo* imaging and treatment [7]. HAP nanostructures could be an ideal candidate for both bioimaging and drug delivery. The investigation on dual or multifunctional HAP systems for biomedical applications has become a trending topic [8].

At present, the rare-earth based inorganic luminescent nanoparticles have gained a lot of popularity because of their potential biomedical applications. The most important applications could be found on pharmaceutical industry or biological and medical diagnostics. A luminescent agent, in this case europium, which has great biocompatibility, is ideal for implantation, imagenology, and clinical application [9]. The doping of materials is a technique that consists of incorporate impurities in the crystal structure of other materials. The doping of hydroxyapatite is possible because, as is known, the europium chemical reactivity is similar to that of calcium [10]. Ciobanu et al. [11] reported the synthesis of doped hydroxyapatite nanoparticles synthesized at low temperature with the atomic ratio Eu/(Ca + Eu) = 1%, 2%, 10%, and 20% and ellipsoidal morphology. Yang et al. [12] synthesized nanosized particles with multiform morphologies via a simple microemulsion-mediated process assisted with microwave heating and reported that the morphologies and the particle sizes of the made samples can be tuned by altering the pH values in the initial solutions. On the other hand, Graeve et al. [13] prepared europium-doped hydroxyapatite and calcium-deficient hydroxyapatite by combustion synthesis and obtained samples with similar crystallite size, particle size, and morphology but the luminescence behavior was different among samples. Han et al. [14] synthesized europium-doped hydroxyapatite by ultrasound assisted precipitation method; their results showed that the luminescence of Eu:HAP was enhanced by the thermal treatment and the increment in Eu content. Escudero et al. [15] prepared hydroxyapatite doped with europium and functionalized them with poly(acrylic acid) PAA following a one-pot microwave-assisted hydrothermal protocol at 180°C which results in a novel morphology for this system. They obtained polycrystalline nanoparticles and showed a spindle-like shape with main dimensions of 191 × 40 nm. Although some europium-doped hydroxyapatite nanoparticles have been reported, these materials have not been really tested against oral fibroblasts (HGF-1) and HeLa cells and as chemotherapy drugs release systems to demonstrate their potential application. Chen et al. reported the synthesis of theranostic Eu^3+^/Fe^3+^ dual-doped hydroxyapatite nanoparticles without a high temperature calcination and with excellent fluorescent properties but they did not test these particles against oral cells [16].

As reported and discussed by Perera et al., synthesis nanoparticles by coprecipitation method without high temperature calcination have attracted more attention for preparing nanohydroxyapatite; in this review, Perera et al. mention several works reporting the synthesis of apatite materials doped with rare earths with excellent fluorescent properties but with micron sizes due to the high calcination temperatures needed to obtain crystalline powders [17]. The microwave-assisted synthesis is an excellent option to overcome the use of a high temperature calcination process but still there is a need for a simpler process [18].

5-Fluorouracil (5FU) is an antineoplastic agent with a relatively short (10–20 min) plasma half-life and commonly used in the therapy of different solid tumors due to its biopharmaceutical and pharmacological properties [10]. It belongs to the class of cytotoxic anticancer drugs that possesses detrimental side effects of attacking both healthy and cancerous cells, which have inhibited their use in spite of its effectiveness towards the destruction of cancer cells [10].

The main objective of this study was to synthetize europium-doped nanohydroxyapatite using a simple aqueous precipitation method and then characterize and impregnate selected samples with 5-fluorouracil in order to explore the properties and releasing capacity of this material. The prepared nanomaterial was characterized using X-ray diffraction analysis (XRD), transmission electron microscopy (TEM), energy dispersive X-ray spectroscopy (EDS), Fourier transform infrared spectroscopy (FTIR), and photoluminescence (PL). Viability and drug release test were performed using oral fibroblasts and HeLa cells.

## 2. Materials and Methods

### 2.1. Synthesis of Hydroxyapatite Nanoparticles

The nanoparticles were synthesized by a wet-chemical precipitation method. To achieve this, 50 mL of a 0.3 M solution of ammonium dihydrogen phosphate [NH_4_H_2_PO_4_] was added dropwise under magnetic stirring to 50 mL of a 0.5 M of calcium nitrate tetrahydrate [Ca(NO_3_)_2_-4H_2_O] with different amounts of europium (III) nitrate hydrate [EuN_3_O_9_-H_2_O] (for more details, see Table 1). Once ammonium dihydrogen phosphate was completely added, ammonium hydroxide solution [NH_4_OH] was added to raise the pH to 10. The precipitate formed was then aged 24 hours and washed five times with deionized water to remove all undesired constituents. The nanoparticles were dried at 80°C during 24 hours and then thermally treated in an autoclave at 120°C for another 3 hours. The precipitate was dried at 80°C for yet an additional 24 hours to finally obtain a white powder.

### 2.2. Characterization

#### 2.2.1. X-Ray Diffraction (XRD)

XRD patterns were recorded using powders and with a GBC-Difftech MMA diffractometer operated in integration mode. The patterns were scanned in the 2*θ* range of 10–80°, with a step size of 0.02°. The nickel filtered Cu K*α* (*λ* = 1.54 Å) radiation was used at 34.2 mA and 35 kV. Rietveld refinement was performed for all samples using MAUD software [19].

#### 2.2.2. Transmission Electron Microscopy (TEM) and Elemental Analysis (EDS)

The specimen for TEM (JEOL JEM-1230) imaging was prepared from the nanoparticles suspension in deionized water. A drop of nanoparticles solution was placed on a 200-mesh copper grid, followed by drying the sample at ambient conditions before it is attached to the sample holder on the microscope and observed at an accelerating voltage of 100 kV.

#### 2.2.3. Fourier Transform Infrared Spectroscopy (FTIR)

The functional groups present in the powder were identified by FTIR (Shimadzu, IRaffinity-1). A certain amount of the nanopowder was collocated in the equipment and the spectrum was taken in the range of 400–4000 cm^1^ with a resolution of 2 cm^−1^ and 200 times scanning using the attenuated total reflection (ATR) method.

#### 2.2.4. Photoluminescence (PL)

PL was observed using a USB4000 spectrophotometer (Ocean Optics); powder samples were excited with a solid state continuous laser (532 nm, 100 mW) using optic fiber.

### 2.3. Cell Culture

Fibroblasts cells (HGF-1) were cultured in a humidified chamber using DMEM supplemented with 20% of fetal bovine serum (10% for HeLa cells) at 37°C and 5% carbon dioxide. Cells were trypsinized at 80% of confluence using a buffered saline solution containing trypsin. Then, the cells were transferred to a 96-well culture plate at 1 × 10^5^ cells per well and incubated for 24 hours to allow attachment [20].

#### 2.3.1. Cell Viability (MTT Assay)

The viability of fibroblasts cells was assessed using the MTT assay. Briefly, the fibroblasts were treated with HA and HAPEu suspensions at different concentrations by 24 and 48 hours in triplicate. HeLa cells were treated only with HAPEu5%+5FU by 24 and 48 h. Thereafter, the reconstituted MTT was added in an amount equal to 10% of the culture medium volume. The culture was returned to the incubator for 4 hours. After the incubation period, the culture was removed from the incubator and the resulting formazan crystal was dissolved by adding an equal amount of MTT solvent as the original culture medium volume. Finally, the optical density was measured using a microplate reader at 570 nm (iMark, Microplate Absorbance Reader, Bio Rad).

### 2.4. Preparation of Drug Storage/Release System

5-Fluorouracil was selected as the model drug. 0.5 g of HAPEu5% sample was added, at room temperature, to 50 mL of PBS solution with a fluorouracil concentration of 250 mg/L, and soaked for 24 hours with continuous stirring. The fluorouracil-loaded HAPEu5% sample was separated by centrifugal action and then dried at 80°C for 24 h. Then, the powder was compressed in disks (0.300 g each, diameter 10 mm) using a pressure of 2 MPa. In the drug release experiments, each disk was immersed into 50 mL of PBS at 37°C with oscillating shaking. 2 mL of the solution was withdrawn for UV-Vis absorption spectroscopy analysis at 265 nm at given intervals to measure the amount of 5-fluorouracil released.

## 3. Results and Discussion

### 3.1. Characterization

**Figure 1:**
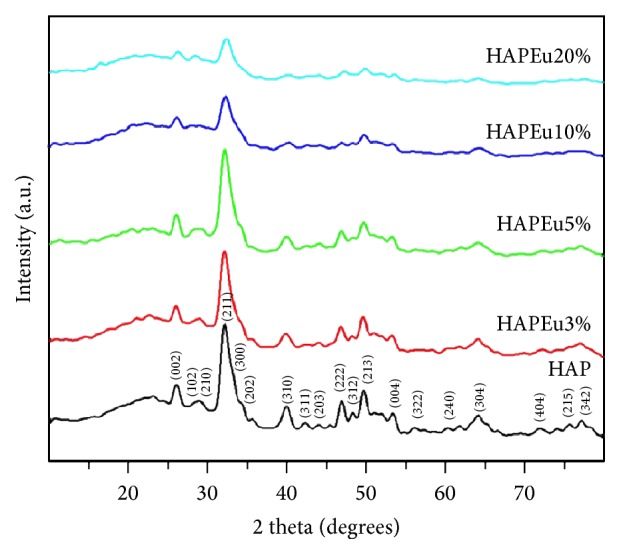
X-ray diffraction patterns of the pure HAP and Eu-doped HAP. Samples can be indexed as pure hexagonal phase (P6_3_/m space group).

Figure 1 shows the XRD patterns of pure HAP and HAPEu samples. The typical diffraction peaks of HAP were found; they can be indexed as the standard data (JCPDS number 09-0432) and as pure hexagonal phase (P6_3_/m space group). In HAPEu samples, the characteristic diffraction peaks of HAP are still present; in general terms, these data show a decrement on the intensities when the concentration of europium increased, suggesting that doping inhibits the HAP crystal growth. The results of the Rietveld refinement are presented in Table 2; as we can see, the incorporation of europium atoms to the crystal structure of HAP modifies the cell parameters as expected; there is a decrease in the value of the cell parameter c while the variations in the value of a are random.

It is a general tendency in our work that the intensity of the XRD peaks decreases with the quantity of europium added; perhaps this tendency is not well appreciated between samples with Eu at 3% and 5% because the difference in the quantity of Eu is not big enough, but the tendency is still present. Ciobanu et al. [11] reported similar results when preparing europium-doped hydroxyapatite nanocrystalline powders and reported that samples with 0.01 and 0.02 of europium are similar, from the XRD point of view, to the undoped hydroxyapatite; they also found that the relative intensities of the peaks decrease with the increment of the europium concentration (0.1, 0.2). On the other hand, Yang et al. [12] found that the relative intensities of the diffraction peaks increase with the increasing of the pH values in the synthesis method (7, 9, and 12); this relation can be due to the enhanced crystallinity of the samples. In our work, we proposed a pH value of 10 because it allows us to obtain nanopowders with an optimal crystallinity, shape, and size. A Rietveld analysis was performed.

**Figure 2:**
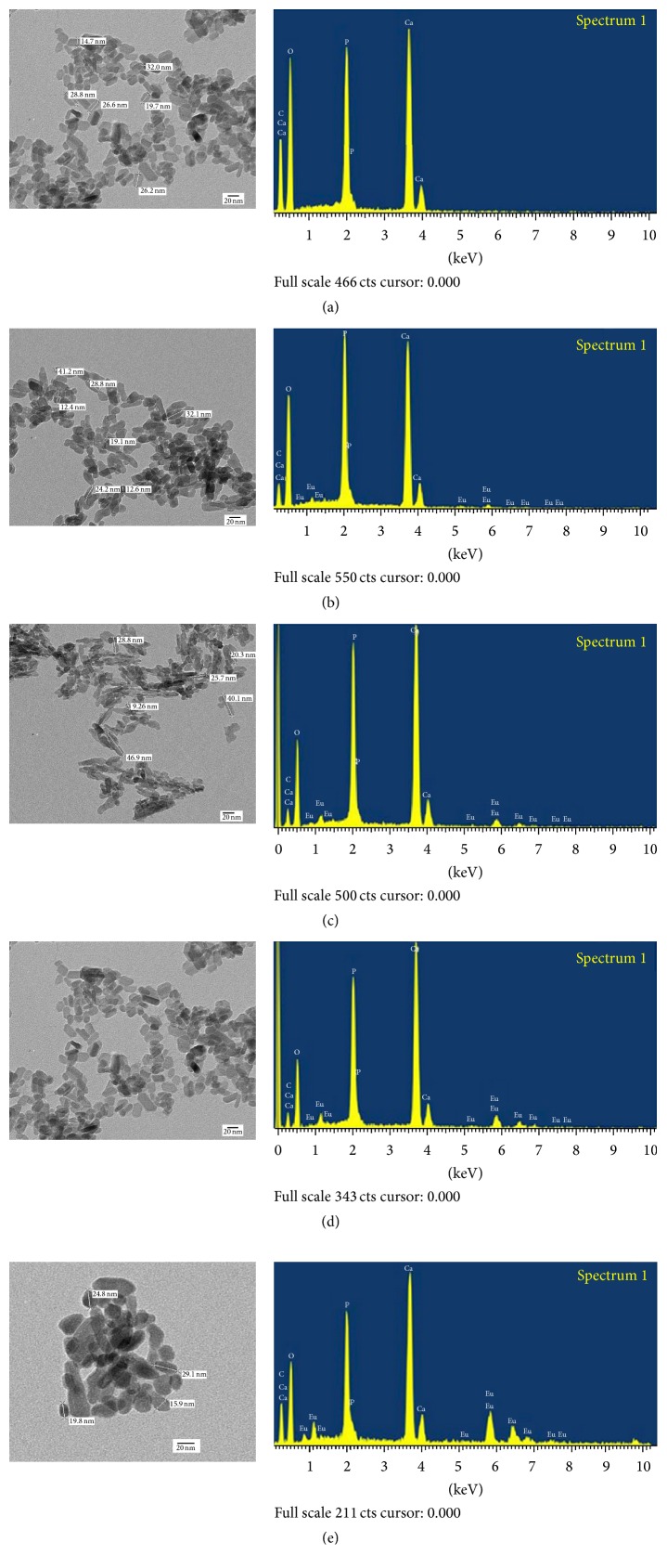
TEM and EDS spectroscopy. (a) Pure HAP, (b) HAPEu3%, (c) HAPeu5%, (d) HAPEu10%, and (e) HAPEu20%. Sizes are estimated to be around 10–50 nm.

TEM images can be observed in Figure 2. The pure HAP (Figure 2(a)) consists of a variety of morphologies and a size of 10–50 nm. HAPEu nanoparticles (Figures 2(b), 2(c), 2(d), and 2(e)) show a rod-like morphology, with a size of 10–50 nm. It is important to mention here that the hydrothermal treatment given to our nanoparticles gives them a well-defined size and morphology. André et al. [21] obtained HA- and Eu-doped HA powders at room temperature and submitted them to the microwave hydrothermal method treatment (HTMW) at 140°C for 0, 1, 20, or 40 min and found a phase with hexagonal structure; they also verified that the HTMW treatment at 140°C at different times improves the crystallization process of the HA compared with samples obtained at room temperature. The use of the hydrothermal treatment to improve or to change the morphology of hydroxyapatite nanoparticles has also been reported by Xuan et al. [22]; they changed a long nanowire morphology with length up to 500 nm and diameter less than 50 nm to a nanorod-like morphology. Silva et al. [23] reported nanoparticles around 27 nm without thermal treatment and 163 nm when a temperature of 1200°C was applied; however, the shape is irregular with or without the thermal treatment.

The easier synthesis method that we propose allows us to obtain hydroxyapatite nanoparticles with a size and shape that are more suitable for biomedical applications (the cellular uptake requires sizes under 70 nm and spherical shape) [16].

The EDS spectrum (Figures 2(b), 2(c), 2(d), and 2(e)) confirms the presence of Ca, P, and Eu. The complete EDS results are shown in Table 1; we can appreciate that the (Ca+Eu)/P relation is close to the range reported for calcium-deficient hydroxyapatite [24].

**Figure 3:**
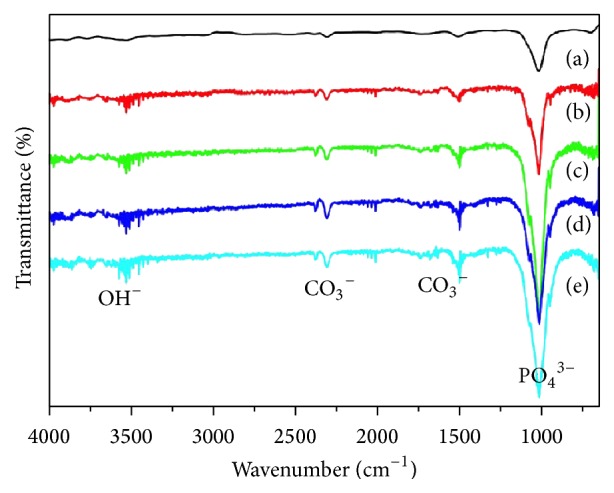
FTIR spectra of the samples prepared in this work. (a) HAPEu20%; (b) HAPEu10%; (c) HAPEu5%; (d) HAPEu3%; (e) HAP.

The FTIR spectra for HAP and HAPEu samples are displayed in Figure 3. All samples exhibited absorption bands at 3570 cm^−1^ due to the -OH stretch. Bands in the range 1000–1100 cm^−1^ indicate the characteristic molecular structures of the polyhedrons of PO_4_^3−^ in the apatite lattice. The CO_3_^2−^ signal can be observed in 1527 and 2300 cm^−1^ bands; its presence is due to the adsorption of atmospheric CO_3_^2−^ during the ripening time. This phenomenon can be attributed to the highly alkaline conditions in the solution, in which there are enough OH^−^ ions for the reaction with CO_3_^2−^. Iconaru et al. [25] reported similar bands around 1090 and 1040 cm^−1^ that can be attributed to the PO_4_^3−^ group; moreover, they observed that the contribution of the area that corresponds to the phosphate bands decreases when the europium concentration in their samples increases.

**Figure 4:**
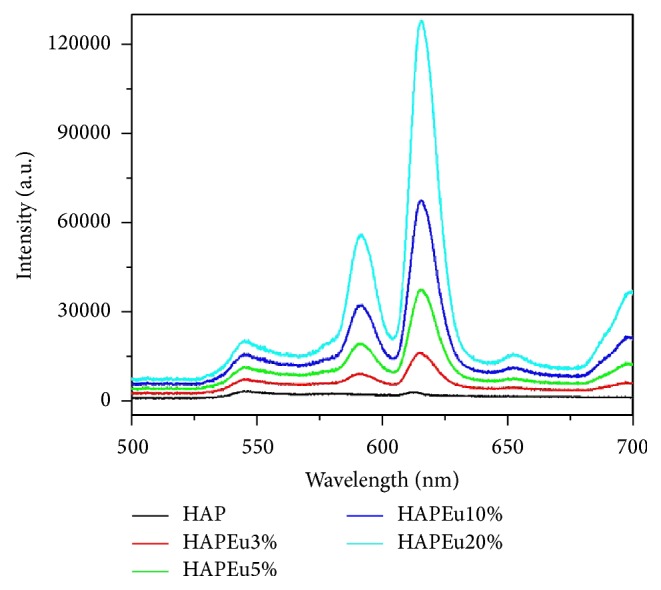
Photoluminescence of HAP and HAPEu nanoparticles measured at room temperature. Excitation source was at 532 nm.

Figure 4 displays the photoluminescence of nanoparticles measured at room temperature. Four emission peaks appeared at about 590, 615, 650, and 699 nm. It can be observed that the photoluminescence increases with the concentration of Eu^3+^. As expected, the HAP sample does not have photoluminescence properties. Sun et al. [26] reported similar results; their samples exhibit the two emission peaks at 592 and 617 and found that the PL intensity increased with the increase in the molar ratio of Eu/(Eu + Ca) to 0.5%, 1%, and 5%, and no significant modification of emission spectra was observed. Chen et al. [5] investigated the effect of the concentration of Eu^3+^ and Gd^3+^ and their experiments showed that the concentration of the dopants had little effect on the emission peaks, but the PL intensity changed by varying the concentrations. Four emission peaks appeared at about 590, 615, 650, and 699 nm at an excitation of 394 nm. The PL emission intensity increased as the concentration of Eu^3^ was increased.

### 3.2. MTT Assay

**Figure 5:**
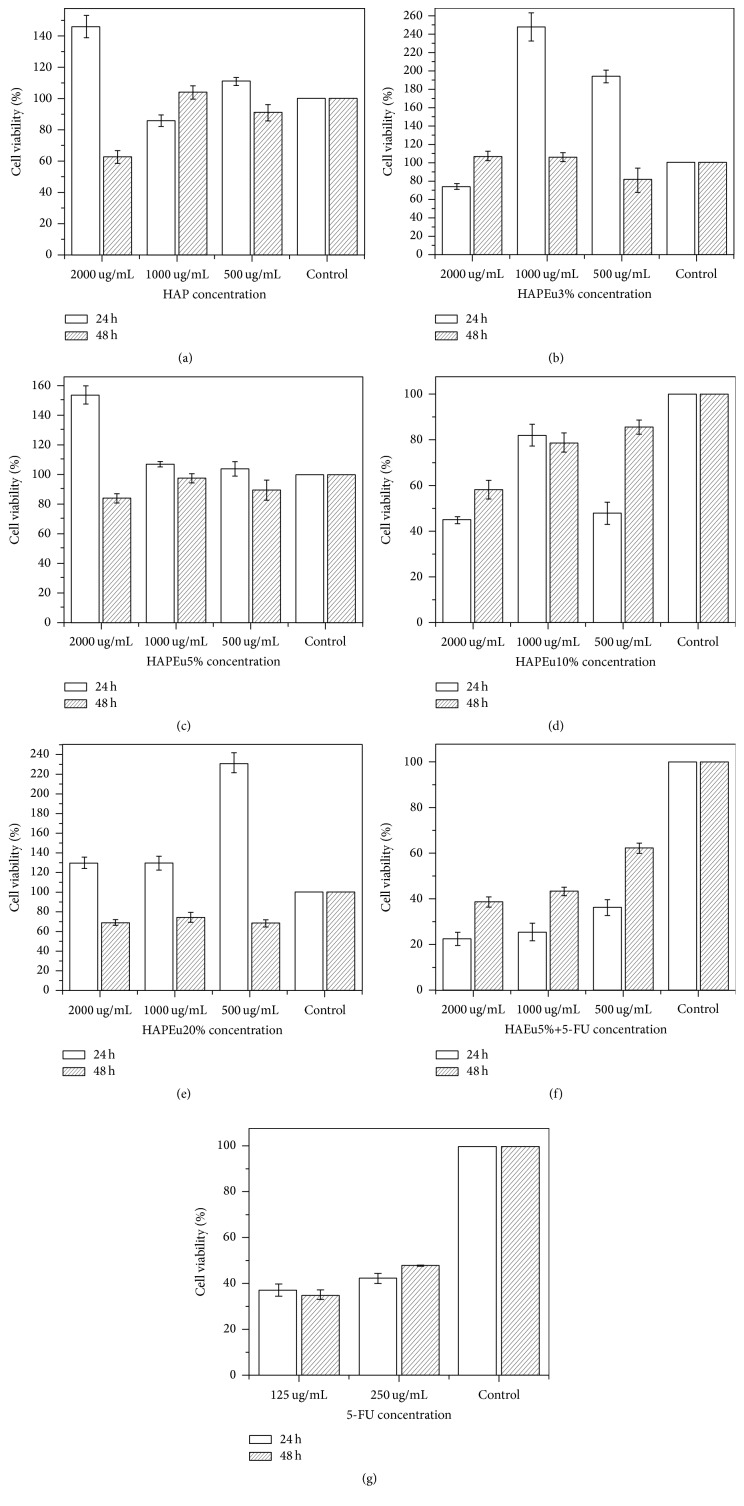
Viability results of the test made using the MTT assay on fibroblast cells (HGF-1). Control is (HGF-1) without sample. (a) Sample HAP; (b) sample HAPEu3%; (c) sample HAPEu5%; (d) sample HAPEu10%; (e) sample HAPEu20%. (f) Results of the viability tests made using the MTT assay on HeLa cells with sample HAPEu5%+5FU. Control is HeLa cells without HAP sample. (g) Results of viability tests using the MTT assay 5-fluorouracil (5-FU) against HeLa cells.

**Figure 6:**
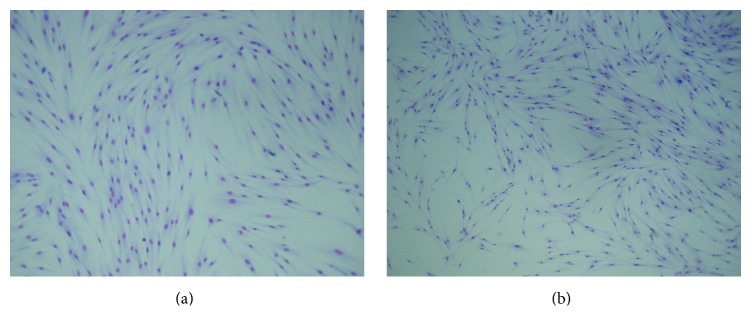
Optic microscope images of oral fibroblast (HGF-1) treated with (a) HAP and (b) HAPEu10% nanoparticles. After their treatment, no changes in morphology and size are appreciated; however, a decreased viability was observed. Treatment conditions are the same as those describe in Section 2.3.1.

Figure 5 shows results from the MTT assays. Fibroblasts were incubated with the HAP sample and with the five different HAPEu samples at 500, 1000, and 2000 *μ*g/mL. MTT assay was assessed after 24 and 48 hours of incubation. The experiments showed (Figure 5) that the viability was the lowest in the HAPEu10% sample at 24 and 48 hours. On the other hand, the cytotoxicity HAPEu5% sample was lower compared with the rest of the samples at 24 and 48 hours and with similar results versus the control group; that is, the cytotoxicity is very low for this sample. These results allowed us to decide the use of HAPEu5% sample for the experiment with 5FU.  Figure 6 shows the morphology of oral fibroblasts (HGF-1) treated with HAP (Figure 6(a)) and HAPEu10% nanoparticles. After their treatment, no changes in morphology and size are appreciated. Viability results in this work are based on the MTT assay; this assay is related to metabolic changes in cells.  Figure 6 reports changes in cell morphology; these techniques are complements and do not always agree; this could be the reason why the effect of HAPEu10% on cell viability was not observed in Figure 6. These results are different from those reported by Naderi et al. [27]; they tested different concentrations of nanohydroxyapatite from 2 to 0.002 mg/mL on gingiva-derived fibroblast cell line (HGF-2) at 24, 48, and 72 hours; they concluded that after 24 hours high doses of nanohydroxyapatite have cytotoxic effect on gingival-derived fibroblasts; our samples are less potential even at high doses of the material. HeLa cells were incubated only with HAPEu5% and 5FU due to their lowest cytotoxicity, at concentrations of 500, 1000, and 2000 *μ*g/mL. The same protocol was carried out as the fibroblast cells, and we observed a reduction in viability in the first 24 hours and after that time an increase at 48 h. Wei et al. [28] agree with our results in the fact that HAP nanoparticles not only inhibit the proliferation but also induce apoptosis of HeLa cells and could provide a basis for treatment of tumors. Hu et al. [6] demonstrated the capacity of HAP material to treat tumors in rabbits; our material could be more potential in this sort of treatments because it has a lower particle size.

### 3.3. Release of 5FU

**Figure 7:**
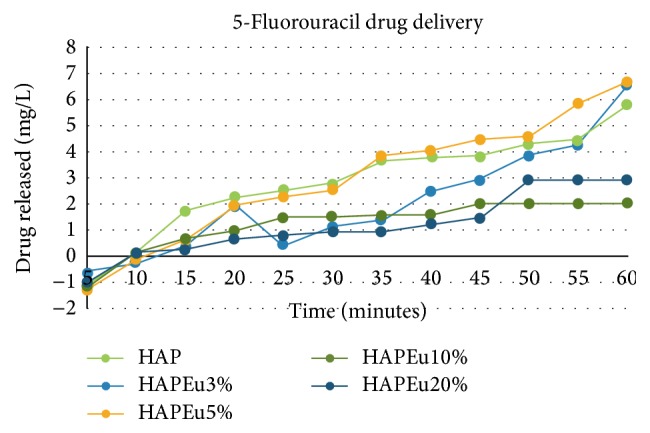
Drug release profile in PBS of HAP and HAPEu samples.

The drug release behavior in PBS of 5FU impregnated in HAP and HAPEu was also investigated.  Figure 7 shows the drug release curves of two drug delivery systems (tablets) in PBS during one hour. It can be seen that HAP and HAPEu5% have the upper release rate of 5-fluorouracil. The drug release behavior of microsized granules having a donut shape at 80°C has been reported and reveals a fast releasing rate of 5FU when soaked in buffer phosphate solution maintained at physiological temperature (37°C); however, the particle presented less crystallinity compared with our synthesis and the drug delivery capacity of our sample HAPEu5%+5-FU is better [10]. Ji et al. [29] also reported the synthesis of hydroxyapatite nanorods and obtained a good drug delivery system because the 5-fluorouracil release time was prolonged to 24 hours, which is much longer than the biological half-life of 10–20 min of the drug; however, when testing the material against bone marrow stem cells, it turned out to be highly toxic when concentrated making them a more potential material than ours.

## 4. Conclusions

We reported a simple aqueous chemical method to prepare Eu-doped hydroxyapatite powders with good crystallinity, a narrow size distribution, and particle size in the range of 10–70 nm; these nanoparticles present strong fluorescence with lines at 590, 615, 650, and 699 nm. The prepared samples show a low cytotoxicity on oral fibroblast cells after 24 and 48 hours of incubation; the viability was the lowest in the HAPEu10% sample. The HAPEu5%+5FU drug delivery system reduces the viability to 20% of HeLa cells after 24 h of contact. The material has good behavior when releasing 5-fluorouracil (5FU) drug. These unique properties make it an ideal material for possible oral biomedical applications.

## Figures and Tables

**Table 1:** EDS results of the samples prepared in this work.

Sample label	Added europium (%wt)	Ca content (at%)	P content (at%)	Eu content (at%)	Ca+Eu/P ratio
HAP	0	6.96	5.14	0	1.35
HAPEu3%	3	9.96	8.11	0.16	1.25
HAPEu5%	5	13.67	10.61	0.69	1.35
HAPEu10%	10	14.44	11.03	1.43	1.44
HAPEu20%	20	10.38	7.32	2.60	1.78

**Table 2:** Rietveld analysis results.

	Refined cell parameters		
Sample	a (Å)	c (Å)	Crystallite size (nm)
HAP	9.4224	6.8928	159.85
HAPEu3%	9.4168	6.8898	133.29
HAPEu5%	9.3771	6.8636	156.58
HAPEu10%	9.3533	6.8644	237.75
HAPEu20%	9.3638	6.7994	176.84
